# Concurrent management of HIV and malaria: A comprehensive review of strategies to enhance quality of life

**DOI:** 10.1097/MD.0000000000037649

**Published:** 2024-04-05

**Authors:** Emmanuel Ifeanyi Obeagu, Getrude Uzoma Obeagu, Nwanganga Ihuoma Ubosi, Ijeoma Chinwe Uzoma, Eltayeb Mohamed Ahmed Tayrab

**Affiliations:** a Department of Medical Laboratory Science, Kampala International University, Kampala, Uganda; b School of Nursing Science, Kampala International University, Kampala, Uganda; c Department of Public Health Sciences, Faculty of Health Sciences, National Open University of Nigeria, Jabi, Abuja, Nigeria; d Molecular-Hematology and Immuno Genetics Unit, Department of Medical Laboratory Science, Faculty of Health Sciences and Technology, University of Nigeria, Nsukka, Enugu State, Nigeria; e Chemical Pathology, Department of Pathology, Faculty of Medicine and Dentistry, Kampala International University, Kampala, Uganda.

**Keywords:** ART, enhance quality of life, HIV, infection, malaria, management

## Abstract

The co-occurrence of human immunodeficiency virus and malaria presents a complex medical scenario, significantly impacting the quality of life for affected individuals. This comprehensive review synthesizes current knowledge, challenges, and strategies concerning the concurrent management of these infections to improve overall well-being. Epidemiological insights reveal the prevalence and demographic trends, highlighting geographical areas of concern and socioeconomic factors contributing to the burden of co-infection. Pathophysiological interactions elucidate the compounding effects, altering disease progression and treatment outcomes. Healthcare challenges underscore the necessity for integrated care models, evaluating existing healthcare frameworks and their efficacy in addressing dual infections. In-depth analysis of interventions explores pharmacological, behavioral, and preventive measures, evaluating their efficacy and safety in co-infected individuals. Additionally, the review assesses psychosocial support mechanisms, emphasizing community-based interventions and peer networks in enhancing holistic care. Consideration is given to the role of antiretroviral therapy, malaria prevention strategies, and the evolving landscape of healthcare delivery in optimizing outcomes for this vulnerable population. The paper concludes by emphasizing the significance of multidisciplinary approaches and integrated care models, stressing the need for continued research and collaborative efforts to advance interventions and improve the quality of life for those navigating the complexities of human immunodeficiency virus and malaria co-infection.

## 1. Introduction

The coexistence of human immunodeficiency virus (HIV) and malaria poses a formidable challenge in the realm of global health, significantly impacting the quality of life and health outcomes for affected individuals.^[1]^ Both infections, prevalent in many parts of the world, especially in regions with limited resources, contribute extensively to the burden of disease.^[2]^ Their concurrent presence amplifies the complexities of healthcare management, necessitating a comprehensive understanding of their interactions and tailored strategies to enhance the well-being of those affected.^[3]^

HIV, a chronic viral infection affecting the immune system, and malaria, a parasitic disease transmitted through mosquitoes, individually pose significant health threats.^[4]^ However, when these infections converge within the same individual, they create a synergistic health challenge.^[5]^ This convergence alters the immune landscape and exacerbates the pathophysiological effects, leading to intricate clinical manifestations and treatment complexities.^[6]^

Understanding the epidemiology and burden of HIV-malaria co-infection is imperative in addressing the unique healthcare needs of this vulnerable population.^[7]^ Geographical hotspots and demographic trends reveal the disproportionate impact of this co-infection, particularly in resource-constrained settings where these diseases often coexist.^[8]^ The intricate interplay between HIV and malaria at a pathophysiological level further complicates disease management.^[9]^ The impact of one infection on the progression, severity, and treatment response of the other necessitates a nuanced approach to healthcare delivery. This includes a deeper exploration of immune dysregulation, treatment interactions, and the implications for long-term health outcomes.

Healthcare systems face multifaceted challenges in managing dual infections, ranging from diagnostic complexities to ensuring equitable access to appropriate treatments.^[10]^ The need for integrated care models that bridge the gap between HIV and malaria healthcare programs becomes increasingly evident.^[11]^ Understanding the effectiveness of existing healthcare frameworks and identifying gaps in service provision is crucial in optimizing care for individuals affected by both diseases. This paper seeks to elucidate these complexities by examining interventions, treatment strategies, psychosocial support mechanisms, and integrated care models aimed at enhancing the quality of life for individuals navigating the dual challenges of HIV and malaria co-infection.^[12]^ By synthesizing current knowledge and evaluating the efficacy of various approaches, this review aims to contribute to the ongoing discourse, fostering advancements in healthcare practices and interventions for improved outcomes in this vulnerable population.^[13]^

## 2. Epidemiology and burden of co-infection

The epidemiology and burden of co-infection involving both HIV and malaria represent a complex and multifaceted challenge in global public health.^[4]^ Understanding the prevalence, distribution, and demographic factors surrounding this co-occurrence is crucial in designing effective interventions and healthcare strategies. Epidemiological data reveals the varying prevalence rates of HIV-malaria co-infection in different regions worldwide.^[14]^ Certain areas, particularly in sub-Saharan Africa and parts of Southeast Asia, experience a higher incidence of both diseases, often overlapping geographically. These regions, characterized by limited resources and high disease prevalence, face a compounded burden due to the coexistence of HIV and malaria. Variations in prevalence rates exist not only between countries but also within different populations and demographic groups, emphasizing the need for targeted interventions tailored to specific contexts.^[15]^ Demographic factors such as age, gender, socioeconomic status, and access to healthcare services significantly influence the prevalence and impact of co-infection.^[16]^ Vulnerable populations, including pregnant women, children, and individuals with limited access to healthcare resources, bear a disproportionate burden.^[17]^ Understanding these demographic trends is crucial for designing inclusive and equitable healthcare strategies that address the needs of diverse populations affected by HIV-malaria co-infection.

The co-occurrence of HIV and malaria strains healthcare systems, affecting diagnostic capabilities, treatment protocols, and resource allocation.^[18]^ Co-infected individuals often present with complex clinical manifestations, complicating accurate diagnosis and necessitating tailored treatment approaches.^[19]^ Additionally, the burden imposed on healthcare infrastructure in regions with a high prevalence of both diseases requires integrated care models that address the simultaneous management of HIV and malaria.^[20]^ Socioeconomic determinants such as poverty, inadequate sanitation, limited access to education, and healthcare disparities contribute significantly to the burden of co-infection.^[21]^ These factors intersect, creating a cycle that exacerbates the vulnerability of certain populations to both HIV and malaria. Addressing these underlying socioeconomic disparities is integral to mitigating the impact of co-infection and improving health outcomes.

Comprehensively understanding the epidemiology and burden of HIV-malaria co-infection involves analyzing prevalence rates, geographic distribution, demographic trends, and the socio-economic factors shaping its impact. This knowledge serves as a foundational framework for developing targeted interventions, allocating resources effectively, and implementing inclusive healthcare strategies aimed at mitigating the burden of co-infection and improving the quality of life for affected individuals.

## 3. Pathophysiology and clinical implications

The pathophysiology of concurrent HIV and malaria infections involves intricate interactions between the 2 diseases, resulting in complex clinical manifestations and implications for disease progression and management.^[22]^ HIV and malaria interact at an immunological level, influencing the body’s immune response. HIV compromises the immune system by targeting CD4+ T cells, impairing their function and reducing the body’s ability to fight infections.^[23]^ Malaria, on the other hand, triggers a robust immune response, leading to inflammation and immune activation.^[24]^ This interplay between HIV-induced immunosuppression and malaria-induced immune activation can exacerbate the severity and complications of both diseases.

Co-infection with HIV and malaria can lead to altered disease progression and increased severity of both illnesses.^[25]^ HIV-infected individuals are more susceptible to severe malaria due to their compromised immune status, leading to higher parasite levels and more severe clinical outcomes.^[26]^ Conversely, malaria can accelerate the progression of HIV by increasing viral replication and reducing the effectiveness of antiretroviral therapy (ART), ultimately impacting the control of HIV infection.^[27]^ The coexistence of HIV and malaria poses challenges in treatment strategies. ART used in HIV management may interact with antimalarial drugs, impacting their efficacy or leading to potential adverse effects.^[28]^ Similarly, drugs used to treat malaria may have interactions with HIV medications, requiring careful consideration and monitoring to avoid adverse outcomes and ensure optimal treatment for both infections.^[29]^

Co-infected individuals may exhibit a wide spectrum of clinical manifestations, ranging from more severe malaria symptoms to atypical presentations of HIV-related illnesses.^[30]^ Complications such as anemia, neurological disorders, and increased susceptibility to opportunistic infections are more prevalent in co-infected individuals, necessitating vigilant clinical management and tailored treatment approaches.^[31]^ The combined impact of HIV and malaria co-infection on disease outcomes can result in poorer prognosis and increased mortality rates compared to individuals affected by either disease alone.^[25]^ The complex interactions between these infections can complicate clinical management, leading to longer recovery times and increased healthcare resource utilization. Understanding the pathophysiological interactions between HIV and malaria is essential for guiding clinical management strategies, optimizing treatment approaches, and developing interventions that effectively address the unique challenges posed by co-infection. This knowledge underscores the importance of integrated healthcare models and multidisciplinary approaches in managing individuals affected by both diseases concurrently.

## 4. Healthcare challenges and integrated care models

The coexistence of HIV and malaria presents significant challenges for healthcare systems, necessitating innovative approaches and integrated care models to effectively address the complex needs of individuals affected by both diseases.^[18]^ Accurate diagnosis of HIV-malaria co-infection requires sophisticated testing methodologies.^[32]^ Challenges arise due to overlapping symptoms, potential interactions between diagnostic assays, and the need for specialized testing facilities.^[33]^ Developing streamlined diagnostic algorithms that efficiently detect both infections is crucial for timely and accurate management. Managing co-infection involves navigating potential interactions between ART for HIV and antimalarial drugs.^[34]^ Selecting appropriate medications that effectively treat both diseases while considering drug interactions, potential side effects, and dosage adjustments is essential.^[35]^ Healthcare providers must receive training and support to make informed decisions regarding treatment regimens for co-infected individuals.

Regions burdened by both HIV and malaria often face resource constraints, including limited healthcare facilities, trained personnel, and medical supplies.^[36]^ Integrating services for HIV and malaria within existing healthcare systems is vital to optimize resource utilization and provide comprehensive care.^[37]^ Strengthening healthcare infrastructure and improving access to essential medicines and diagnostic tools are critical components of effective management.^[38]^ Implementing integrated care models that bring together HIV and malaria services streamlines patient care, improves treatment adherence, and enhances health outcomes.^[39]^ Co-location of services, such as combined clinics or coordinated care pathways, allows for holistic management, including prevention, diagnosis, and treatment of both infections. This approach facilitates collaboration between healthcare providers, promotes efficient information sharing, and enhances patient-centered care.

Engaging communities and providing education about co-infection, its implications, preventive measures, and treatment options are integral components of successful healthcare strategies.^[40]^ Empowering communities to actively participate in their healthcare fosters early detection, treatment adherence, and reduces stigma associated with co-infection.^[41]^ Continuous training and capacity building among healthcare professionals are essential to equip them with updated knowledge, skills, and tools necessary for managing co-infection effectively. Providing specialized training in integrated care approaches and multidisciplinary collaboration enhances the quality of care delivered to co-infected individuals. Addressing healthcare challenges associated with HIV-malaria co-infection requires a multifaceted approach that encompasses improvements in diagnostics, treatment strategies, healthcare infrastructure, integrated care models, community engagement, and ongoing capacity building.^[42]^ Integrated care models that consider the complex interactions between HIV and malaria are pivotal in ensuring comprehensive and effective management while optimizing health outcomes for individuals affected by both infections.^[39]^

## 5. Interventions and treatment strategies

Interventions and treatment strategies for managing concurrent HIV and malaria infections involve a multifaceted approach aimed at addressing the unique challenges posed by co-infection.^[43]^ These strategies encompass pharmacological, preventive, and behavioral interventions tailored to optimize outcomes for individuals affected by both diseases. Ensuring access to effective ART for HIV-positive individuals is crucial in managing co-infection.^[44]^ Optimizing ART regimens that consider drug interactions with antimalarial treatments and potential side effects is essential. Regular monitoring of viral load and CD4+ cell counts helps gauge the effectiveness of HIV treatment in co-infected individuals.^[45]^

Deploying effective antimalarial treatments and preventive measures is vital in co-infection management.^[46]^ Utilizing artemisinin-based combination therapies for malaria treatment while considering dosage adjustments in individuals receiving ART is important.^[47]^ Additionally, implementing preventive measures like insecticide-treated bed nets and indoor residual spraying can reduce malaria transmission among co-infected populations. Implementing integrated healthcare models that combine HIV and malaria services streamlines care delivery.^[48]^ Co-location of services, joint consultations, and shared patient records improve treatment adherence, reduce healthcare fragmentation, and enhance patient outcomes.

Focusing on preventive measures to reduce the risk of HIV transmission among individuals with malaria, such as promoting safe sex practices, providing education on HIV prevention, and offering pre-exposure prophylaxis for at-risk populations, can significantly impact the spread of HIV in co-infected settings.^[49]^ Routine screening for both HIV and malaria in endemic areas facilitates early detection and timely initiation of treatment. Integrated testing approaches and point-of-care diagnostics improve accessibility and facilitate prompt management of co-infection.^[50]^ Promoting behavior change through health education programs encourages adherence to medication, adoption of preventive measures, and early healthcare-seeking behavior. Empowering individuals with information about co-infection, its management, and the importance of treatment adherence enhances patient engagement in their healthcare. Continued research into novel therapies, diagnostic tools, and vaccine development for both HIV and malaria is crucial.^[51]^ Innovation in healthcare technologies and interventions can drive advancements in co-infection management, improving treatment efficacy and outcomes. Tailoring interventions and treatment strategies to the specific needs of individuals affected by both HIV and malaria is essential.^[52]^ These multifaceted approaches, encompassing medication optimization, preventive measures, integrated service delivery, education, and ongoing research, form the foundation for effective co-infection management and improved quality of life for affected individuals.

## 6. Psychosocial and community support

Psychosocial and community support play a pivotal role in addressing the holistic needs of individuals affected by concurrent HIV and malaria infections.^[53]^ These supportive interventions aim to improve mental health, social well-being, and overall quality of life for co-infected individuals and their communities. Providing access to mental health professionals, counselors, and support groups can help individuals cope with the emotional stress, anxiety, and depression associated with co-infection. Psychosocial support services offer a safe space for co-infected individuals to express their concerns, receive counseling, and learn coping strategies to manage the emotional impact of living with both diseases.

Community-based education programs that dispel myths, reduce stigma, and raise awareness about HIV and malaria co-infection are crucial. Educating communities helps foster understanding, empathy, and acceptance, reducing discrimination and social isolation faced by co-infected individuals.^[54]^ Establishing peer support networks allows individuals living with co-infection to connect with others facing similar challenges. Peer support fosters a sense of belonging, reduces isolation, and provides a platform for sharing experiences, knowledge, and practical advice on managing the diseases. Empowering individuals through health literacy initiatives enables them to make informed decisions about their healthcare.^[55]^ Providing information on disease management, treatment adherence, and available support services empowers co-infected individuals to actively participate in their care and advocate for their needs.

Engaging communities in designing and implementing support programs fosters ownership and sustainability.^[56]^ Community involvement ensures that interventions are culturally sensitive, contextually relevant, and responsive to the unique needs of the population affected by co-infection.^[57]^ Economic empowerment programs, vocational training, and income-generating activities can alleviate financial burdens and improve the socioeconomic status of co-infected individuals. Stable livelihoods and economic opportunities contribute to improved well-being and better access to healthcare. Strengthening family and social support systems provides a crucial safety net for co-infected individuals. Support from family members, friends, and the community creates a conducive environment for adherence to treatment, reduces social isolation, and promotes overall well-being. Designing interventions that respect cultural norms, beliefs, and traditions enhances their acceptability and effectiveness within communities.^[58]^ Culturally sensitive approaches ensure that psychosocial and community support interventions are relevant and inclusive. Psychosocial and community support initiatives are integral components of holistic care for individuals living with concurrent HIV and malaria. By addressing emotional, social, economic, and cultural aspects of co-infection, these interventions contribute significantly to enhancing the overall quality of life and well-being of affected individuals and communities.^[59]^

## 7. Conclusion

The management of concurrent HIV and malaria infections presents a multifaceted challenge that significantly impacts the health and quality of life of affected individuals. Addressing the complexities of co-infection requires a comprehensive and integrated approach that encompasses various aspects of healthcare, psychosocial support, community engagement, and education. Throughout this review, the epidemiological landscape, pathophysiological interactions, healthcare challenges, interventions, and support strategies have been highlighted. The prevalence of co-infection in specific regions, especially in resource-limited settings, emphasizes the need for targeted interventions and integrated care models tailored to diverse demographic groups.

The intricate immunological interplay between HIV and malaria underscores the need for optimized treatment approaches, careful consideration of drug interactions, and the development of innovative therapies. Integrated healthcare models that combine services for HIV and malaria streamline care delivery, improve treatment adherence, and optimize health outcomes for co-infected individuals. Psychosocial and community support interventions play a pivotal role in addressing the holistic needs of co-infected individuals, focusing on mental health, stigma reduction, empowerment, and community involvement. These initiatives not only improve emotional well-being but also contribute to better treatment adherence and overall health outcomes. Moving forward, a concerted effort from healthcare systems, policymakers, researchers, community organizations, and affected individuals is imperative. Collaboration and innovation in healthcare delivery, sustained investment in healthcare infrastructure, and a commitment to addressing social determinants of health are crucial in improving the lives of individuals affected by HIV and malaria co-infection.

## Author contributions

**Conceptualization:** Emmanuel Ifeanyi Obeagu.

**Methodology:** Emmanuel Ifeanyi Obeagu, Getrude Uzoma Obeagu.

**Supervision:** Emmanuel Ifeanyi Obeagu.

**Validation:** Emmanuel Ifeanyi Obeagu.

**Visualization:** Emmanuel Ifeanyi Obeagu.

**Writing – original draft:** Emmanuel Ifeanyi Obeagu, Getrude Uzoma Obeagu, Nwanganga Ihuoma Ubosi, Ijeoma Chinwe Uzoma, Eltayeb Mohamed Ahmed Tayrab.

**Writing – review & editing:** Emmanuel Ifeanyi Obeagu, Getrude Uzoma Obeagu, Nwanganga Ihuoma Ubosi, Ijeoma Chinwe Uzoma, Eltayeb Mohamed Ahmed Tayrab.
